# Thyroid Cancer After Chernobyl: Re-Evaluation Needed

**DOI:** 10.5146/tjpath.2020.01489

**Published:** 2021-01-15

**Authors:** Sergei Jargın

**Affiliations:** Department of Pathological Anatomy, Peoples’ Friendship University of Russia, Moscow, Russian Federation

**Keywords:** Ionizing radiation, Chernobyl, Cancer risk, Thyroid cancer

## Abstract

Thyroid carcinoma in people exposed to radiation during their childhood and adolescence is the only solid cancer for which the incidence increase as a result of the Chernobyl accident is regarded to be proven. The main evidence in favor of a cause-effect relationship between radiation and thyroid cancer incidence increase comes from epidemiologic studies. Bias in some studies was caused by the screening effect, improved diagnostics after the accident, overdiagnosis, registration of patients from non-contaminated territories as Chernobyl victims, recall bias, dose-dependent selection and self-selection. Prior to the accident, the registered incidence of pediatric thyroid carcinoma was lower in the former Soviet Union than in other industrialized countries i.e. there were undiagnosed cases in the population. The screening found not only small nodules but also late-stage tumors interpreted as radiogenic cancers developing after a short latency. Pediatric thyroid cancers detected during first 10 years after the accident were larger than those detected later on average, many tumors being poorly differentiated and metastatic. The relationship of thyroid cancer and Chernobyl exposures is not denied here; however, it is argued that the quantity of radiogenic cases has been overestimated according to the mechanisms discussed in this paper. In addition, it is suggested that results of some Chernobyl-related molecular-genetic and other studies should be re-evaluated, considering that many tumors detected by the screening or brought from non-contaminated areas and registered as exposed to the fallout were advanced cancers.

## INTRODUCTION

This is an update and continuation of the article previously published in the Turkish Journal of Pathology (1). A significant increase in the thyroid carcinoma (TC) incidence occurred after the Chernobyl accident (CA) among people exposed as children and adolescents. There has been no convincing evidence of a cause-effect relationship between radiation exposures from CA and incidence increase of other solid cancers (2,3). The dramatic elevation of TC incidence after a short latent period came unexpectedly upon the scientific community (4–6). The consequences of CA could not be predicted from studies of atomic bomb survivors (3). Medical exposures to radioiodine were not convincingly associated with an increased TC risk (7–13). The main evidence in favor of a cause-effect relationship between ionizing radiation and TC incidence rise after CA came from the epidemiologic research. Confounding factors and bias in some studies have been discussed previously: the screening effect, high alertness, overdiagnosis, registration of non-exposed patients as Chernobyl victims, recall bias, dose-dependent selection, and self-selection (14–16). Apparently, the quantity of radiogenic TC cases has been overestimated according to the mechanisms discussed below.

### Pediatric TC Before and After the Chernobyl Accident

Prior to CA, the registered incidence of pediatric TC had been considerably lower in the former Soviet Union (SU) than in other industrialized countries (17,18). In the 1981-1985 period, the TC incidence among children ≤15 years old in the northern regions of Ukraine was 0.1 and in Belarus it was 0.3 per million per year (18). For comparison, the US Cancer Registry reported the total incidence rate for the period 2000-2004 equal to 85 per million per year, ~2.1% being diagnosed at the age ≤20 years. According to the Tumor Registry in Germany, the incidence was 69 in adults, 0.2 in children 0-9 years old, 0.4 in those aged 10-14 years, 1.4 in adolescents 15-19 years old and 2.0 per million per year in total for those ≤20 years (19). The TC incidence grew with advancing age also in later reports from the United States: 0.43 (5-9 years old) to 3.5 (10-14 years) and 15.6 (15-19 years) per million per year (20,21). Of note, the TC incidence in Belarus in people ≤18 years old has remained at the enhanced level (15.7 per million per year in 2012) (22,23), although the radiation factor has no longer been active, which indicates that other mechanisms e.g. enhanced vigilance have contributed to the high figures. It had been known prior to CA that screening can significantly elevate the detection rate of TC (24).

The comparatively low detection rate of TC prior to CA is often disregarded in the literature. It was claimed, for example, that the frequency of sporadic TC in Belarus during the 1971-1985 period did not differ from the global statistics (25) with reference to (6), where no such statements were found. Balonov (26) wrote about the background TC incidence in children ≤10 years old of 2-4 cases per million per year in Belarus and Ukraine, which disagrees with the data cited above (18). The relatively low TC frequency in the contaminated areas prior to CA indicates that there were neglected cancers among residents. The screening after CA found not only small nodules but also late-stage TC interpreted as rapidly growing radiogenic cancers developing after a short latent period. Besides, there was endeavor to be recognized as Chernobyl victims to gain access to health care and other provisions (27). Cases from non-contaminated areas must have been averagely higher-grade as there was no mass screening there. Accordingly, the “first wave” TC cases after CA were larger and of higher grade than those diagnosed later (28), when neglected cases had been sorted out by the screening. Pediatric TCs detected during first 10 years after CA were described as relatively low differentiated, aggressive, invasive and metastatic (29). It can be argued that the screening cannot account for differences in the patients’ age as a significant increase occurred only in people exposed as children and adolescents. The mechanism was selection bias: children were given more attention, and they are accessible for screening at schools and preschools; mass checkups were performed in conditions of high alertness. As mentioned above, screening can significantly elevate the registered TC incidence (24) due to a “reservoir of clinically silent cancers” (30). Obviously, mechanisms such as the counting of tumors with uncertain malignant potential and microcarcinomas among cancers, false-positivity, and the registration of non-exposed patients as radiation-exposed have contributed to the incidence rise. The relatively high prevalence of latent thyroid microcarcinomas in the population is known; as discussed previously, some of these cases have been overtreated (31). The following statement can cause misunderstanding: “77% of primary tumors were larger than 1 cm, suggesting that these were not incidental TC detected by screening” (32). In fact, the screening detected not only small nodules but also advanced TC, neglected because of the incomplete coverage of residents by medical checkups prior to CA. This predictable phenomenon was confirmed by the fact that the “first wave” TC cases after CA were on average larger and higher-grade than those found later (28).

Considering the misinterpretation of late-stage TC as aggressive tumors caused by radiation, some features of supposedly radiogenic cancers must characterize, on average, a later stage of the tumor progression. For example, chromosomal rearrangements of the proto-oncogene *Ret*, especially *Ret/PTC3* fusions, frequently found in TC of patients exposed to radiation after CA at a young age (5,33), were supposed to be markers of radiogenic TC (34,35). In fact, as discussed previously, the *Ret/PTC3 *frequency among TC patients probably correlates with the average disease duration and tumor progression (36). The cohort of pediatric papillary TC after CA with prevailing *Ret/PTC3*, detected during the first 10 years after CA, was deemed exceptional worldwide (37,38). In sporadic papillary TC, *Ret/PTC1 *fusions are more frequent than for *Ret/PTC3* (39). In fact, post-Chernobyl cancer is exceptional not worldwide but in more developed countries, where malignancies are detected relatively early. Similarly to the former SU, *Ret/PTC3* was the most prevalent *Ret* fusion type among TC cases from India (40). In particular, *Ret/PTC3* fusions were reported to be frequent in Kashmir (41). On the contrary, pediatric TC in Japan has been different from that after CA, being averagely of lower grade (42). The *Ret* fusions in the pediatric TC in Japan were found only in ~10.3%; expectedly, *Ret/PTC1* was the prevailing *Ret *fusion type (43,44). This certifies the relatively early diagnostics of TC in Japan. Along the same lines, more mutations were found in TC from contaminated areas of Russia compared with controls from Seattle (45). Additional details are summarized in several previous papers (36,46).

No associations between *Ret/PTC* and radiation doses were reported in a research of nodular thyroid lesions in the areas of Russia contaminated after CA (47). Correlations between individual doses and *Ret/PTC* among atomic bomb survivors (48) could have been caused by a bias similar to that discussed here as well as by higher doses. Note that for the low-LET (linear energy transfer) radiation, acute exposures are generally more efficient than the same doses protracted over a long time; an overview of literature was provided previously (49). The enhanced frequency of *Ret/PTC* was reported in papillary carcinomas from patients who had undergone radiotherapy in childhood. Many of these patients had been treated for cancer so that the doses were comparatively high (50). The possibility that mutations such as *Ret/PTC* can be induced by radiation is not denied here. Of importance is the accumulation of mutations in parallel with the tumor dedifferentiation and their association with certain steps of the neoplastic progression, *Ret/PTC3* - with a later step than *Ret/PTC1* (36,46).

### Epidemiological Studies

The main body of evidence in favor of the cause-effect relationship between ionizing radiation and TC among children and adolescents after CA has come from the epidemiologic studies e.g. those regarded pivotal (16,51–54). In the case-control study (52), a retrospective estimation of doses was carried out by means of questionnaires. The research by Davis et al. (53) was similar in design. The “Chernobyl victim syndrome” (27) was a widespread phenomenon: many patients strived for higher dose estimates to support their status of Chernobyl victims and could provide biased information. Moreover, cancer patients tend to recollect circumstances related to the exposure better than controls (55). The low participation rate among controls was of concern because of potential selection bias (52). Furthermore, no widespread prophylaxis by stable iodine occurred in the most contaminated areas of Belarus and the Russian Federation immediately after the accident. The measures were taken months after the accident to provide stable iodine to children (52). Nonetheless, the iodine supplementation was reported to reduce the cancer risk approximately threefold (52), although there would be no appreciable blockage of the radioiodine uptake by the thyroid (7). Other questionable aspects of the study design (52), favoring an LNT-type dose-response relationship, have been commented on previously (56).

Cohort studies applied interviews along with thyroid dosimetry to estimate individual doses. Dosimetry was performed within 2 months after the accident (t1/2 of 131I is about 8 days). The study design included, if indicated, repeated examinations in central clinics of Kiev or Minsk (16,54). It can be reasonably assumed that persons with higher dose estimates would be more interested in further examinations on average. In the health care system of the former SU, the thoroughness of medical examinations sometimes depended on the patient’s initiative. The dose-dependent participation of cases could have resulted in higher estimates of risk (16). Other epidemiologic studies on the effects of low-dose low-rate radiation may be laden by the same bias and others (24,57). Of note, a significant increase of benign thyroid nodules was found in individuals exposed as children or adolescents to the Chernobyl fallout (58–60). The pathogenesis of benign lesions is different from that of papillary TC (61). The commensurate frequency increase of both benign and malignant thyroid nodules is circumstantial evidence in favor of the role of non-radiation factors. Additional details are summarized in the previous paper (1).

Furthermore, detection of thyroid cancer is heavily dependent on the intensity of screening, which can elevate the detection rate manifold (24,62). The screening effect, improved registration and other non-radiation-related factors have played their role in the post-Chernobyl incidence increase of TC (4). Radio- and cancerophobia contributed to the overdiagnosis of cancer, which can be illustrated by the following citation from a Russian-language professional publication (verbatim translation): “Practically all nodular thyroid lesions, independently of their size, were regarded at that time in children as potentially malignant tumors, requiring an urgent surgical operation” (63). Obviously, mass screening in the areas where pediatric TC had been rarely diagnosed before, in the atmosphere of radio- and cancerophobia, must have resulted in overestimation (14,15).

### Some Aspects of Morphological Diagnostics

Mechanisms of the overdiagnosis of TC after CA have been discussed previously (1,15,36,46). If a definite conclusion about malignancy cannot be made on the basis of a fine-needle aspiration (FNA), a histological examination is required. The surgical specimen is forwarded to the department of pathology, where malignancy of a radically removed lesion could have been confirmed also in case of uncertainty, favored by the insufficient quality and quantity of histological specimens. Cases of false-positive diagnosis, caused by misinterpretation of nuclear atypia as a malignancy criterion of thyroid nodules, are known from practice. The overdiagnosis was favored by high tumor expectancy and limited availability of foreign literature. FNA was started later than ultrasonography, which additionally contributed to the false-positivity during the 1990s. Panel reexaminations with international participation confirmed ~78% of histological diagnoses of post-Chernobyl pediatric TC in Russia. The cytological diagnosis of TC was confirmed histologically in 161 of 238 cases (68%), among them papillary carcinoma in 69.5% (64). The cutting up of surgical specimens was performed in many institutions with blunt knives without access to running water, which may lead to the tissue squashing, displacement of cells and tissue fragments (65). This can explain, for example, the detection of malignant cells within blood vessels in 45% of childhood TC cases (66). During the 1990s, celloidin embedding was still in use, where all nuclei appear somewhat cleared or “ground-glass-like” compared to paraffin-embedded specimens, which can be misinterpreted as a diagnostic criterion of papillary TC. False-positive cases, not covered by reexaminations, remained uncorrected also because histological specimens were not always stored properly, some slides were missing or “taken for consultation” etc. More details and references are in several previous papers (1,64). The misinterpretation of late-stage malignancies as aggressive radiogenic cancers had consequences for the therapy (31).

## CONCLUSION

The above and previously published (1,14,15,36,64,67) arguments cast doubt on the causality between radiation exposures after CA and the increase in the registered cancer incidence. The relationship of TC and Chernobyl exposures is not denied here; however, it is argued that the quantity of radiogenic cases has been overestimated according to the mechanisms discussed in this paper. In the author’s opinion, results of some Chernobyl-related molecular-genetic and other studies should be re-evaluated, considering that many tumors detected by the screening during the first decade after CA or brought from non-contaminated areas and registered as exposed to the fallout were in fact advanced cancers. Accordingly, some supposed markers of radiogenic cancer are probably associated with the disease duration and tumor progression. The exaggeration of Chernobyl consequences is potentially misleading in regard to the carcinogenicity of low-dose low-rate radiation, especially from radioiodine.

The monitoring of populations exposed to low-dose radiation is important but will hardly add much reliable information on the health risks. It can be reasonably assumed that the screening and increased attention of exposed people to their health will result in new reports on the elevated cancer detection rate in exposed populations. An alternative for future work would be large-scale animal experiments. The average life duration is known to be a sensitive endpoint attributable to radiation exposure. Further experiments with different animal species would lead to a better quantification of their radiosensitivity thus enabling more precise extrapolations to humans.

## CONFLICT of INTEREST

The author declares no conflict of interest.
